# The Law of Lunacy in France
*"De l'Interdiction des Aliénés et de l'État de la Jurisprudence en Matière de Testaments dans l'Imputation de Démence." Par A. Brierre de Boismont. Avec des Notes de M. Isambert, Conseiller à la Cour de Cassation. Paris: Baillière. 1852.


**Published:** 1852-10-01

**Authors:** 


### Art. IV.—THE LAW OF LUNACY IN FRANCE.*

The laws of lunacy have been the subject of as much anxiety and deliberation in France as they have in England; and the articles contained in the Code of Interdiction prescribing the circumstances and regulations under which persons accused of being insane may be decreed incapable of managing their affairs, and deprived of personal liberty, have been repeatedly under discussion; and their revision proposed with the view of throwing some additional security round the personal comforts and interests of persons so afflicted. The most able statesman, and the most eminent physicians, have conferred together and united their counsels in endeavouring to accomplish this end; but the wisest system of human legislation, even the laws of Lycurgus, Numa, and Solon, will be found to be defective on certain points; and if it be found so difficult to make laws for the government of a community, the members of which are presumed to be capable of reasoning, and cognizant of their civil responsibilities, how much more perplexing must it be to devise laws for a class of subjects who are placed in a very dubious position, until declared incapable of managing themselves or their affairs, and who are then unhappily too often surrounded by unprincipled relations and pretended friends, who are actuated only by selfish motives. The desecration of the dead is not a more

* "De l'Interdiction des Aliénés et de l'État de la Jurisprudence en Matière de Testaments dans l'Imputation de Démence." Par A. Brierre de Boismont. Avec des Notes de M. Isambert, Conseiller à la Cour de Cassation. Paris: Baillière. 1852.

heinous offence than the robbery of those helpless beings who have outlived the self-protecting powers of their own intellectual faculties. The insane in France are professedly a "State care;" they are so also in England, although less avowedly, inasmuch as the Lord Chancellor is the recognised "Parens Patriæ" on the part of the Crown, and the delegated representative of the reigning sovereign. There is, indeed, a close analogy between the principles and the administration of the laws of lunacy in both countries; as, however, lunatic asylums in France are specially government institutions, so they are organized on a larger scale than they are with us: witness the Charenton, Salpêtrière, Bicêtre; the physicians connected with which have constantly before them an immense field for observation and practice. We therefore take up the works of such men as Pinel, Esquirol, Calmeil, Georget, Falret, Mitivié, Voisin, Leuret, Foville, Baillarger, Brierre de Boismont, &c., with intense interest, and seldom or never lay them down without being gratified by their perusal. There appears, indeed,—and we confess it—to be more activity and greater emulation in pursuing medico-psychological researches abroad than at home, as is evinced by the number of distinguished physicians who contribute regularly to the "Annales Medico-Psychologiques," the "Annales d'Hygiene Publique et de Médecine Legale," and other scientific serials which are constantly emanating both from the French and German press. Among the *élite* of these authors who have devoted special attention to the theory and practice, and all questions connected with insanity, and who have presented many valuable contributions to this department of professional literature, the name of Brierre de Boismont stands pre-eminent. We need scarcely add, that it is well-known to the readers of this journal.

The *brochure* now before us, on the Laws of Interdiction and the state of jurisdiction in France, as affects the wills of persons imputed to be in a state of dementia—to which some notes are appended by M. Isambert, the eminent advocate to the Court of Cassation—contains a recapitulation of some of the most important of these ordonnances, which M. Brierre de Boismont contends are not framed with due regard to the present advanced state of medical knowledge. It sets out with condemning the limited application of the 489th Article of the Code, which provides that "A person of age, who is in a state of habitual imbecility, dementia, or furor, may be interdicted, even though lucid intervals do occur in this state." But before entering upon the legal merits of the points which he selects for disquisition, M. Brierre de Boismont lays before us some psychological views respecting the nature of insanity which claim our attention.

The difficulty of giving a logical definition of terms, the signification of which is nevertheless commonly understood, is well known to every student in philosophy; and the impossibility, we fear, of logically defining forms of mental disease which are nevertheless self-evident in their manifestation, gives rise, both in civil and criminal cases, to a vast amount of learned quibbling and ingenious sophistries. Fencing with shadows is a judicial art. Courts of justice are not temples for the enunciation of simple truths; as such they cannot be at once received, but must be assayed in a variety of ways; the Genius of the Law must sift and melt them down in its own crucible, and after confusing the elements together, will then set about separating them, for the purpose of bringing into evidence as much of the pure ore as it may be convenient to separate from its own adventitious and alloying dross. Hence, before such tribunals expert counsel belonging to a profession which is, in every direction, fettered with technicalities, shine to advantage in demanding medico-psychological definitions which they are aware cannot easily or readily be given. "Can the medical witness not favour the court with an explication of words which he must know are commonly used on such subjects?" "Can he not define logically [by laying down the *genus* and *differentia*] according to the rules of logic, the species of insanity which he describes?" We fear not; and have a notion that Polonius was a true philosopher when, with becoming brevity, he said to Hamlet's mother, the Queen of Denmark,

"Your noble son is mad!
*Mad!* call *I* it; for to *define* true madness,
What is 't, but to be *nothing else than mad!*"

Certain it is, that all definitions of insanity, whether proposed by psychologists or physicians, have hitherto been unsatisfactory, and the one suggested by M. Brierre de Boismont is, in its turn, not less so. He proceeds on the assumption that insanity consists essentially in a disease of the reasoning faculty; wherefore, to form a just criterion, or test of the existence of mental alienation, we should acquire "la connaissance exacte de la raison;" but how often, as M. Brierre de Boismont well knows, do we find insane persons reason with marvellous critical *acumen* on a vast variety of subjects; sometimes even on points connected with their own peculiar delusions. Hence Locke observed, that "madmen do not appear to have lost the faculty of reasoning; but having joined together some ideas very wrongly, they mistake them for truths, and they err as men do that argue right from wrong principles."—("Essay on the Human Understanding," vol. i. c. ii.) "If the definition," says M. Brierre de Boismont, "which I have given be correct, we shall have a foundation upon which to proceed in demanding an interdiction, or establishing the nullification of certain acts;" but we confess that he gives a wider latitude to the operation of the reasoning faculty than we are inclined to adopt. "Essayons" (we quote his own words, as being important to the argument) "de résumer ce qu'il y a de plus important sur ce sujet; en principe la raison combine les idées, saisit leurs rapports, formule les jugements, les contrôle, en affirme la rectitude ou la fausseté; aussi est ce à juste titre que ces opérations l'ont fait considérer comme un pouvoir intellectuel." (p. 6.) He then refers to the commonly received division of the mental faculties into those connected with the powers of the understanding or intellect, and those connected with the affective or moral feelings, appetites, passions, affections, and propensities. Whatever may be M. Brierre de Boismont's own psychological theory, we cannot allow that reason is always concerned in the combination and association of ideas, in the perception of their relations, or in the formation of judgment. The faculties of the imagination and memory are obviously often most predominant in combining and associating together ideas, whether suggested by the subjective operations of the mind, or from external impressions conveyed to it through the medium of the senses. We do not here recognise the operation of the reasoning faculty. Again: judgment, we apprehend, is an act of the mind, specifically different from that of reason.

"The power of reasoning," says Reid, with whose philosophical essays M. Brierre de Boismont appears to be acquainted, "is very nearly allied to that of judging, and it is of little consequence in the affairs of life to distinguish them nicely. On this account, the same name is often given to them both. We include both under the name of reason. The assent we give to a proposition is called judgment, whether the proposition be self-evident or derive its evidence by reasoning from other propositions. Yet there is a distinction between reasoning and judging. *Reasoning is the process by which we pass from one judgment to another, which is the consequence of it.* Accordingly, our judgments are distinguished into intuitive, which are not grounded upon any preceding judgment, and discursive, which are deduced from some preceding judgment by reasoning. . . . . Reasoning, as well as judgment, must be true or false; both are grounded upon evidence which may be probable or demonstrative, and both are accompanied with assent or belief. The power of reasoning is justly accounted one of the prerogatives of human nature, because by it many important truths have been and may be discovered, which without it would be beyond our reach; yet it seems to be only a kind of crutch to a limited understanding. We can conceive an understanding superior to human, to which that truth appears intuitively which we can only discover by reasoning. For this cause, though we must ascribe judgment to the Almighty, we do not ascribe reasoning to him, because it implies some defect or limitation of the understanding. Even among men, to use reasoning in things that are self-evident is trifling; like a man going upon crutches when he can walk upon his legs."

We can assure M. Brierre de Boismont that we have no disposition to cavil upon psychological distinctions which have not a practical bearing; but were we to admit the criterion he has laid down, that the sanity of the human mind should be tested by the integrity of the reasoning faculty, we should be necessarily obliged to repudiate the existence of all those forms of insanity in which the reasoning powers remain unimpaired, however misguided they may be by erroneous impressions consequent upon insane illusions. The inevitable consequence of his doctrine leads M. Brierre de Boismont himself, upon the very threshold of his argument, to confound together the two great elementary divisions of the mind into intellectual and moral faculties; he tells us, the distinction between the intellectual and moral powers does not really hold good. "Si l'analyse," he observes, "distingue ces deux éléments, l'observation prouve qu'ils ne sauraient être *séparés*." Whatever may be the classification of the faculties of the mind which psychologists may have adopted,—and the most common division recognised by the ancients was under the two general heads of the powers of the understanding and the powers of the will,—these faculties were never presumed to be so many distinct and separate entities, capable of acting independent of each other; but they have always been regarded as links of the same chain—elements of the same intellectual system. We apprehend, however, that M. Brierre de Boismont interprets too freely the meaning both of Reid and Condillac in the passages he has quoted, for the purpose of showing that they agreed in the absolute unity of the intellectual and moral faculties. What is the language of Reid on the subject? "As the mind exerts some degree of activity even in the operations of understanding, so it is certain that there can be no act of will which is not accompanied with some act of understanding. The will must have an object, and that object must be apprehended or conceived in the understanding. It is therefore to be remembered, that in most, if not in all, the operations of the mind, both faculties concur, and we range the operation under that faculty which hath the larger share of it."—(Intellect. Powers. Essay I. chap. vii.) In the passage quoted by M. Brierre de Boismont from Jouffroy's translation, the English philosopher observes—"The faculties of the understanding and of the will are easily distinguished in thought; but it happens very seldom, if ever, that they are disjoined in operation. In the greater number of the operations of the mind, perhaps in all, the two faculties intermix, and we are at the same time intelligent and active."—(Brochure, p. 7.) Here, however, it is clear that Reid does not, as M. Brierre de Boismont interprets, affirm the unity of the intellectual and moral faculties; he recognises their separate existence, and tells us only, that they act co-ordinately, and we "range the operation under that faculty which has the largest share in it." Neither does the quotation from Condillac support the hypothesis. "If we would," he observes, "appreciate the mind properly, it is not enough to analyze the operations of the understanding, but those of the passions also which combine together under the same cause," which we may readily admit without assenting to M. Brierre de Boismont's inference, that would annihilate the identity and distinction which may clearly enough be drawn between the intellectual and moral faculties. All that Reid and Condillac could possibly mean is, that these different faculties act, as we have repeatedly observed in this journal, "in and through each other," as they are as much parts of the same system as the different levers and wheels of a steam-engine are parts of the same machine, the operations of which being combined, produce a given effect. They may nevertheless be separately recognised, and each *per se* identified; hence the different intellectual faculties are subject to specific lesions—perception, memory, judgment, imagination, may each in its turn be affected, and give a morbid colouring or tone to the mind in its unital condition. The faculties of perception may, from physical causes affecting the brain, be deranged, and the judgment, unimpaired, continue to be capable of correcting its false impression, as was the case with Nicolai, who reasoned against the existence of the spectral illusions which he beheld. Illusive appearances are frequently so impressed on the retina, and these false perceptions upon which persons themselves reason at the time they exist are well-known pathognomonic signs of many diseases, which affect by sympathy the organs of sense. Again, the memory may be affected in such a variety of ways, that an account of the lesions to which it is subject would carry us far beyond our present limits. Suffice it therefore to observe, that the intellectual faculties are subject to their own specific aberrations, which must be well known to M. Brierre de Boismont, as well as to all physicians who are engaged in the practice of insanity. What is true as affects the faculties of the understanding, is equally true as affects the moral and affective faculties; the appetites, affections, and passions of our nature are equally subject to perversion and derangement, albeit the faculty of reasoning and the other powers of the understanding may remain unimpaired. Such cases come under the head of Moral Insanity. "It is indeed strange," says Sacasse, the eminent French advocate, "that the moral faculties of an individual shall be deranged, yet the intellect retain its normal state of activity." It may be so; but facts, irrefragable facts, observed by Pinel, Esquirol, Prichard, and others, clearly prove that such may be the case. The intellectual faculties—"the powers of perception and imagination," observes Pinel, "are frequently disturbed without any excitement of the passions;" and, on the other hand, he adds, "the functions of the understanding are perfectly sound, while the man is driven by his passions to acts of turbulence, outrage, and insanity." We therefore differ from M. Brierre de Boismont, who argues that the application of the criminal and civil code of insanity should be founded on the recognition of the principle that an integral unity exists between the intellectual and moral faculties : we hold, on the contrary, that these faculties, although co-operating and blending together, are so many distinct powers, differing in their modes of operation, and subject each in its turn to characteristic aberration; but as the mind can only be occupied with one idea at one time, it is as a *whole* affected when under the influence of any specific lesion. We have dwelt upon this psychological point at some length, because it is of great practical importance in the pathology of those mental diseases which so frequently come under medico-legal discussion.

The laws of interdiction in France have been very carefully and cautiously devised by the legislature ; but it is complained, with justice, that many of the enactments are not sufficiently explicit and comprehensive, and do not come up to the present state of medical knowledge. The 489th Article is complained of as being particularly defective :— "A person of age who is in a state of habitual imbecility, dementia, or furor, should be interdicted, even although lucid intervals may occur in such states." This article suggested a Memoir which M. Brierre de Boismont read about twenty years ago to the Academy of Science:—

"He could not," he informs us, "from his own observations and experience, do otherwise than perceive its extremely limited application, inasmuch as cases of furor may have been common when patients were chained, beaten, and exhibited like wild beasts ; but this state of things no longer exists ; and in well-conducted establishments we seldom witness states of furor excepting as a temporary *passager* symptom of acute mania. The legal signification of the word dementia is very different from that which physicians understand by it as indicating a state of chronic debility, and sometimes rapid failure of the intellect. A very considerable class of insane persons—monomaniacs—are not even mentioned. Whatever latitude may be given to this 489th Article, logically and practically speaking, it would be impossible to include under either of these three denominations that singular aberration of mind which dwells only on one idea—or rather, on a series of ideas—while the person so affected appears to preserve the integrity of his reason on all other subjects."—p. 16.

What becomes of M. Brierre de Boismont's criterion of insanity— the "connaissance exacte de la raison"—which should be the only principle upon which persons should be held amenable to the laws of interdiction?

"The madman (he continues) who imagined that he had a head made of glass, and whom Alexander of Tralles cured by covering it with a helmet of lead, was neither imbecile, demented, nor furious. The same remark applies to the Jesuit, Sgambari, who, supposing himself a cardinal, answered one of the superiors who endeavoured to persuade him of his delusion :—' One of two things must be true ; I am either in my senses, or I am mad ; if I am rational, your language is exceedingly impertinent ; if I am mad, you are more insane than I am, to attempt to convince me by reasoning with me.' These two persons obviously came under that division or form of disease to which the term of monomania has of late years been applied, and which is composed of innumerable kings, poets, sorcerers, imaginary popes, &c.—a variety of insanity observed in the most remote ages ; the only novelty connected with which is the term under which it is now designated—viz. monomania."—*Brochure*, p. 16.

Certainly, it is remarkable that the Code of Interdiction should not have contained any article specifically applicable to a form of disease so frequently observed, and which often very seriously implicates the disposition of property, and the safety of the public ; but such is the force of truth, that notwithstanding the indisposition of the authorities in France to recognise this form of disease, we have two cases published in the *Brochure* before us, one tried before the Tribunal de la Seine, the other before the Court of Appeal at Bourdeaux, in which the validity of two wills was disputed and set aside upon evidence proving the testators, in each case, to have dictated them under the influence of monomania. The observations of M. Brierre de Boismont, and the cases to which he refers, are exceedingly interesting ; but the evidence in proof of the existence of monomania, intellectual and moral, we conceive to be already sufficiently conclusive.

The legislation for the protection or interdiction of the insane upon general principles must necessarily involve many extremely difficult points ; and among these none can be more perplexing even for the physician than to determine the precise period when, during the invasion or early stages of the disease, an individual should be declared within the jurisdiction prescribed by the code. We find persons, before the existence of insanity is suspected, committing eccentric, and, towards themselves, ruinous acts ; squandering away large sums of money, and diverting their inheritance out of its natural channel, away from their own children. Yet the apparent integrity of their intellectual faculties, when such deeds were executed, and such wills drawn out, could not be doubted. Many such cases are given by M. Brierre de Boismont, and in a medico-legal point of view they are exceedingly interesting; but during the stage of *incubation*, a subject to which a few years ago we devoted a special monograph,* and before any *bonâ*

* "On the Incubation of Insanity," by Forbes Winslow, M.D., published in the "Transactions of the Medical Society of London."

*fide* act of insanity has been palpably committed, we do not perceive how any person can properly be declared within the jurisdiction of the law. We may know, we may clearly recognise, the existence of the incipient or rather premonitory symptoms of the disease, which may be visible only to an experienced eye, but until the malady has unequivocally declared itself, such is the jealousy which must ever guard the liberty of the subject, that any interference would be considered premature; and the self-infliction of an individual wrong must be allowed rather than the admission of a principle, the misapplication or abuse of which may peril the rights and liberties of other members of the community. We cannot arm the law with an authority to execute its mandates by anticipation; the evil it contemplates controlling must *de facto* be proved to exist; but the relations or friends of persons who are suffering from an impending attack of insanity, should have recourse to a physician who is conversant with the treatment and pathology of mental diseases, and adopt such measures as he may recommend. Hereby many a sad domestic tragedy now recorded in the annals of domestic history might have been averted; and we feel assured that in many such doubtful and anomalous cases a timely appeal to the physician would save the misfortunes of many private families being exposed in detail before the inquisitorial investigation of a public court. The hand of Humanity would ever willingly draw a veil round the afflictions of the domestic hearth. In all cases where the disease is known to have been hereditary; where symptoms of approaching paralysis, partial or general, are manifest, and where epilepsy has already supervened, the prognosis must be unfavourable; and the presumption is, that sooner or later the laws of interdiction will of necessity be appealed to for the protection of a person so affected.

While the greatest circumspection should be used to protect an individual suspected of being insane, from being prematurely placed under interdiction, so the same amount of care ought to be taken that the legal restriction over his civil rights and liberties should not be continued longer than is necessary. The duration of insanity, its curability or incurability, must in many cases appear very doubtful, and the physician may be called upon to give a prognosis which will be attended with very serious responsibility. Many years ago, an opinion prevailed that this disease was incurable, and doubtless many unfortunate creatures, chained to the walls of their cells in the *oubliettes* of the Bicêtre, and other lunatic asylums, died the victims of this deplorable and cruel ignorance. But since Pinel and Esquirol humanized the treatment of insanity, and enlightened the public mind, this erroneous notion has been exploded; and, according to the calculations of M. Brierre de Boismont, one out of three cases of every form and variety of the disease is now curable. There are many difficulties, we fear, attending an accurate return of the cures in lunacy; and many of the statistical tables published by our own county asylums present us, we suspect, with only approximative results. It is not easy indeed to trace the history of discharged patients, many of whom having been sent away and returned "cured" or "recovered" in the column of the "Register of Discharges," may nevertheless relapse, and be lost sight of even by the Commissioners. The prognosis of the malady being incurable ought, at all events, to be pronounced with very great caution, as is sufficiently proved by many cases to which M. Brierre de Boismont has referred. One of these is reported in a memoir by Dr. Renaudin. A gentleman after being declared incurably insane, perfectly recovered; the interdiction was of course superseded, and upon recovering his liberty, he found that his library and a valuable collection of curiosities, which he had been at great pains in collecting, had been long since sold. Another gentleman, in the prime of life, was placed under interdiction; he remained for several years in an asylum, and eventually recovered. Upon his return home, he found himself completely stripped of all his possessions; his house, furniture, and all his personals, had been sold, and he no longer retained a single acre of land. We hope, under our own system of commissions in lunacy, *we* "manage these things" *better than they* do "in France;" and that the committee of the person and estate, responsible to the Court of Chancery, will be found, in most cases, to exercise the powers delegated to them with discretion and judgment. As some guide to our forming our prognosis, M. de Brierre de Boismont, after alluding to the labours of Séguin, Vallée, Voisin, Belhomme, Guggenbuhl, in ameliorating the condition of idiots and cretins, observes,

"Cases of dementia are reputed to be incurable. Chronic insanity is not easily cured; and the difficulty is increased in proportion as the predisposing causes have been long in existence before the irruption of the disease. When the malady follows general paralysis, scorbutus, epilepsy, and is attended with symptoms of dementia, it is incurable. When the organic functions preserve their wonted integrity, and the patient eats, sleeps, and gains flesh, we may anticipate a cure. When the delusions, however, are very extravagant, or the insane are prompted to eat their own excrements, they are seldom curable. Insanity arising from moral causes, which have been slow in producing the effect, are cured with difficulty; as are also those patients who are led away by religious delusions, self-exaltation, and hallucinations. The insane who can reason upon, and form a pretty correct judgment of, their own condition, are very difficult to cure, unless the return of their rationality be very speedy. (Esquirol.) Insanity arising from intemperance, or the abuse of intoxicating liquors, may be soon cured, particularly in the beginning of the disease, or if it be the first attack. Puerperal mania is of transient duration. Cases of monomania are cured with less facility than those of mania; and monomania, when the patient is cheerful, is more curable than when attended with depression of spirits (*lypemania*). In general, insanity, although hereditary, may be cured; but relapses are to be apprehended. However long may be the period that mental alienation has existed, recovery may still be hoped for; and whenever moral causes act promptly and manifestly, the circumstance augurs well for the cure."—*Brochure*, p. 53.

These conclusions M. Brierre de Boismont has deduced from his own very extensive observations and experience; but as every cas must be judged of by its own peculiar features and progress, it is not easy to lay down any general rules of prognosis. The lucid intervals which sometimes unexpectedly occur, and which are not unfrequently of considerable duration, are very apt to mislead even the experienced physician, who is always naturally anxious to comply with the wishes of the relations and friends of a patient who appears to have recovered his reason, and to be again capable of managing his affairs. Upon this subject the observations of M. Brierre de Boismont are extremely judicious, and lead him to consider the circumstances under which the *main-levee* of the interdiction should be recommended. In such cases the same difficulty exists in France as in England, for experience constantly proves, it is extremely difficult to get the Court of Chancery to supersede a commission in lunacy. The object of the interdiction, as of the commission, is the protection of the lunatic and the management of his property during his temporary incapacity to conduct his own affairs. When, therefore, he has perfectly recovered, and is in a sound state of mind, such restriction should undoubtedly be removed. Lord Eldon, one of the most conscientious and learned judges that ever adorned the bench, observed, that "there was no part of the jurisdiction in lunacy more unpleasant, and requiring greater caution, than that of determining when a commission should be superseded; for though a safe conclusion may, upon evidence, be arrived at in establishing lunacy, it is very difficult to determine when the mind has been restored." The difficulty, however, consists, not in the interpretation or administration of the law, but in proving that the recovery is complete, and likely to continue permanent.

We could obviously avail ourselves of the many interesting and suggestive observations of M. Brierre de Boismont, to prolong our present notice to an almost indefinite extent, but our limits are circumscribed. We dissent, it is true, from some of his psychological views, but these do not affect the results of his experience in the treatment of the disease, nor are such differences in theory any reproach to the study of psychology, or the practical application of its doctrines. There are many diseases of the body the pathological origin of which are as little understood. Who has yet satisfactorily shown the origin of tubercular consumption? Nevertheless, physicians agree upon the principles which should guide them in the treatment of this complaint. It is the same with insanity; our psychological theories may differ, but all physicians who devote themselves to this speciality of the profession are agreed upon the principles which should guide them in the medical and moral treatment of its different forms and varieties. We therefore recommend M. Brierre de Boismont's able pamphlet, "De l'Interdiction des Aliénés," to the attention both of the medical and legal profession; it is replete with facts which will be read with interest.

